# Comparison of Adaptation between the Major Connectors Fabricated from Intraoral Digital Impressions and Extraoral Digital Impressions

**DOI:** 10.1038/s41598-017-17839-4

**Published:** 2018-01-11

**Authors:** Ning Gan, Yaye Ruan, Jian Sun, Yaoyang Xiong, Ting Jiao

**Affiliations:** 1 Department of Prosthodontics, 9th People’s Hospital, Shanghai Jiao Tong University School of Medicine, Shanghai Key Laboratory of Stomatology, Shanghai, PR China; 2 Department of Endodontics, School and Hospital of Stomatology, Tongji University, Shanghai Engineering Research Center of Tooth Restoration and Regeneration, Shanghai, PR China

## Abstract

The objective was to compare the adaptation between the major connectors of removable partial dentures derived from intraoral digital impressions and extraoral digital impressions. Twenty-four volunteers were enrolled. Each volunteer received an intraoral digital impression and one extraoral digital impression digitized from conventional gypsum impression. A software was used to create the major connectors on digital impression datasets. After all the virtual major connectors designed from Group intraoral digital impressions (Group I) and Group extraoral digital impressions (Group E) were directly fabricated by 3D printing technique, the adaptation of the final major connectors in volunteers’ mouths were measured. The adaptation ranged from 159.87 to 577.99 μm in Group I while from 120.83 to 536.17 μm in Group E. The adaptation of major connectors in Group I were found better at the midline palatine suture while the adaptation of major connectors in Group E were found better at the two sides of the palatal vault. In both groups, the highest accuracy in adaptation was revealed at the anterior margin of the major connectors. It is feasible to manufacture the major connectors by digital impression and 3D printing technique. Both the adaptation of the two kinds of digital impressions were clinical acceptable.

## Introduction

CAD/CAM (computer aided designed/computer aided manufactured) technique provides a new way with economized manpower and improved efficiency to manufacture dental restorations. Digital impression serves as the first step of dental CAD/CAM technique, which includes two methods for data acquisition: direct intraoral scanning or indirect extraoral scanning^1^. So far in the fields of CAD/CAM fixed dental prostheses (FDPs), extraoral digital impression by scanning gypsum casts poured from conventional impressions is more often applied in clinical situations for its ability to get rid of the scanning difficulties arising from intraoral conditions such as saliva, blood and limited spacing^2,3^. However, clinical procedure started with conventional impression taking still has not met the goal of complete digitization and automation which is the major trend in dentistry^4^. Direct intraoral scanning is truly free of a physical impression^5^ so that it provides a more time-efficient and more patients-preferred way for data acquisition^6^. Many researches have proven that single crowns and FDPs manufactured from intraoral scanning data can deliver comparative marginal and internal fit with those fabricated from conventional impressions^7–13^.

Comparing much progress of CAD/CAM techniques for teeth-supported restorations with digital impressions, development for removable partial dentures (RPDs) is relatively slow. It is more complicated because RPDs frameworks may not only cover a wider range of dentitions but also soft tissues that covered by major connectors. Major connectors, one of the important components of removable partial dentures, are able to connect the parts on both sides into a whole integrally, delivery and distribute occlusal force to abutment teeth or adjacent supporting tissues, and meanwhile enhance the strength of the removable partial dentures^14^. As touched with soft tissues directly, whether the adaptation of major connectors is good or not will influence wearing comfort and the health of oral mucosa. A previous study reported that it was feasible to use the intraoral scanner to obtain digital impressions for whole upper jaws, including full dentitions and palatal soft tissues^15^. RPD frameworks fabricated from the intraoral or extraoral digital impression datasets showed good adaptation as the results obtained by traditional casting method^16,17^, which supported that digital impressions and CAD/CAM techniques could be an alternative choice for the conventional fabrication of RPD frameworks. But few studies are available regarding the quantitative analysis on the adaptation of CAD/CAM RPDs to guide the clinical application.

Dental CAD/CAM techniques also include another two major parts: designing by software and manufacturing by computer-controlled machines. The latest software such as CEREC 4.4 of Sirona and Dental System of 3 Shape have specific modes of restorations, implantology or orthodontics to help dentists and technicians to make treatment plan and design dental prostheses with great convenience. PolyJet, a kind of three-dimensional (3D) printing technique specialized by Stratasys Corporation, jets and instantly UV-cures tiny drops of liquid photopolymer layer by layer on the tray to create 3D models with no post-curing needed^18^. It creates smooth and accurate models with microscopic layer resolution down to 0.1 mm^18,19^, which is able to reproduce more accurate anatomic details than other common RP techniques^20,21^.

To date, available studies on RPD frameworks manufactured by intraoral digital impression combined with CAD/CAM techniques are still limited^17,22^. This *in vivo* study was designed as a self-controlled experiment. The objective was to compare the difference in intraoral adaptation between the 3D-printed major-connectors of RPDs derived from intraoral and extraoral digital impressions. The null hypothesis was that: (1) it was feasible to manufacture the major connectors of RPDs by intraoral digital impressions and 3D printing techniques; (2) there was no significant difference in adaptation measurement between the major-connectors fabricated from the two different digital impression data.

## Material and Methods

### Participants

The study was registered by the Chinese Clinical Trial Registry (Registration No. ChiCTR-OPN-15005929) and approved by the Independent Ethics Committee of Shanghai Ninth People’s Hospital affiliated to Shanghai Jiao Tong University, School of Medicine (Application No. [2014]81) in December 23, 2014. The study was conducted in accordance with the Declaration of Helsinki. Volunteers who gave informed consent and met the following criteria were enrolled into the study.

The inclusion criteria were (1) Volunteers from Shanghai Jiao Tong University School of Medicine; (2) Aged at least eighteen years; (3) Good oral hygiene; (4) Complete maxillary dental arch except the missing third molar; (5) Intact hard and soft tissues, including treated teeth decay and healed teeth extraction socket. The exclusion criteria were (1) Undergoing orthodontic treatment; (2) With metal crowns and any other metal materials on teeth; (3) Advanced periodontitis affecting gingival recession; (4) Obvious teeth mobility (mobility degree higher than 1); (5) Obvious dentition malalignment (malalignment degree higher than 1).

### Sample Size

The clinically significant level was when the power of the statistical test was larger than 0.9. In this study, α = 0.05, β = 0.1, and 1-β = 0.9. To determine the sample size, prior to this study we conducted preliminary experiments to calculate the sample size using the following equation^23^:

$${\rm{n}}={[\frac{({{\rm{Z}}}_{{\rm{\alpha }}}+{{\rm{Z}}}_{{\rm{\beta }}}){{\rm{\sigma }}}_{{\rm{d}}}}{{\rm{\delta }}}]}^{2}$$

where Z_0.05_ = 1.96 and Z_0.1_ = 1.282. σ_d_ means the standard deviation of the difference value of the matched group (referred to the adaptations of major connectors fabricated from intraoral and extraoral digital impression of the same volunteer). δ means the average value of the difference of the matched group. After the data analysis of preliminary experiments, the mean adaptation of major connectors derived from intraoral digital impressions was (358.22 ± 19.27) μm and the mean adaptation of major connectors derived from extraoral digital impressions was (266.90 ± 19.91) μm. According to the equation above, we calculated that the minimum sample size should be 22 volunteers.

### 3D datasets generation, Design, Manufacture of the Major Connectors and Occlusal Rests

**Figure 1:**
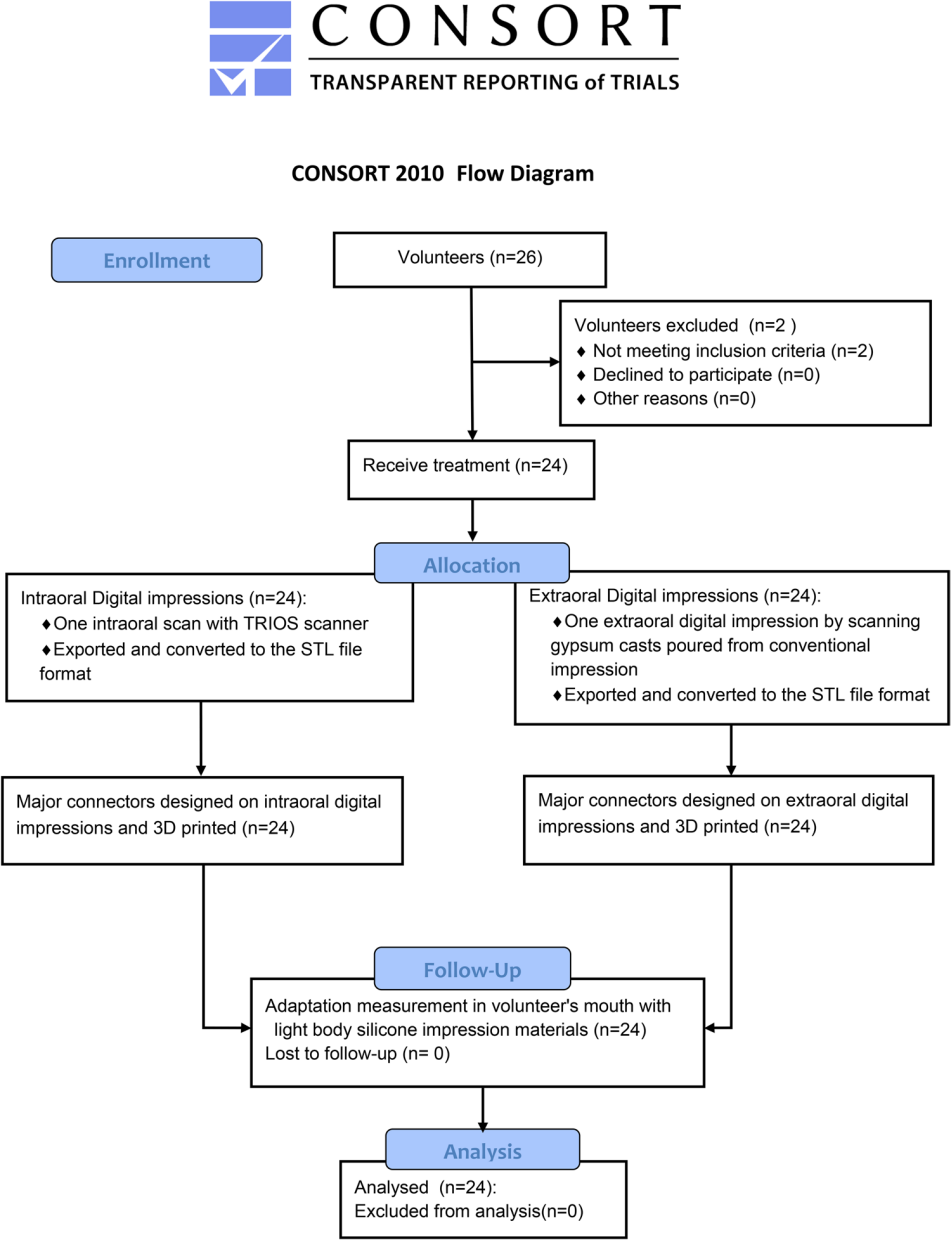
Flow chart. Schulz KF, Altman DG, Moher D, CONSORT Group. CONSORT 2010 Statement: Updated Guidelines for Reporting Parallel Group Randomised Trials. PLoS Med 2010;7(3): e1000251.

The whole enrollment of volunteers and the 3D datasets generation of digital impressions were conducted in the way which was published in our previous study^15^. The volunteers were recruited from January 1, 2015 to June 30, 2015 and the volunteers’ data were collected from February 1, 2015 to July 16, 2015. The whole enrollment and allocation of volunteers are shown in Fig. 1. The first scanning of three intraoral digital impressions was selected as each volunteer’s impression datum in Group intraoral digital impressions (Group I). All the digital impression data of volunteers were imported into the specific design software (Dental System, 3Shape’s 3rd generation Removable Partial Design, 3 Shape, Copenhagen, Denmark), and the major connectors of removable partial dentures, in the form of palatal plates, were designed on the intraoral and extraoral digital impressions respectively. The palatal plate was selected because of its rigidity, support, suitability, patient tolerance^24^, and also convenience in measuring adaptation at different portions of the major connectors. After surveying and blockout of the undercuts of 3D model, the boundary of the major connector was drawn and then the main body of the major connector was generated automatically. Occlusal rests were placed on the mesial-occlusal surface of the first premolars and the distal-occlusal surface of the first molars on the two sides. Minor connectors were placed between the adjacent teeth connecting the occlusal rests and major connectors. The occlusal rests and the minor connectors were smoothed by “Carve” tool. Finally, four support rods with the diameter of 2.5 mm were placed across corners and at the midpoints of the anterior and posterior borders. It was much easier to determine the anterior boundary of the major connector because there were palatal rugae serving as references. To make sure the two major connectors of each volunteer had the common posterior boundaries to the greatest extent, the function of distance measurement in 3Shape design software was used to calculate the length between the anterior and posterior ones. If needed, it would be returned to former procedures to adjust the location of boundaries accordingly.

**Figure 2:**
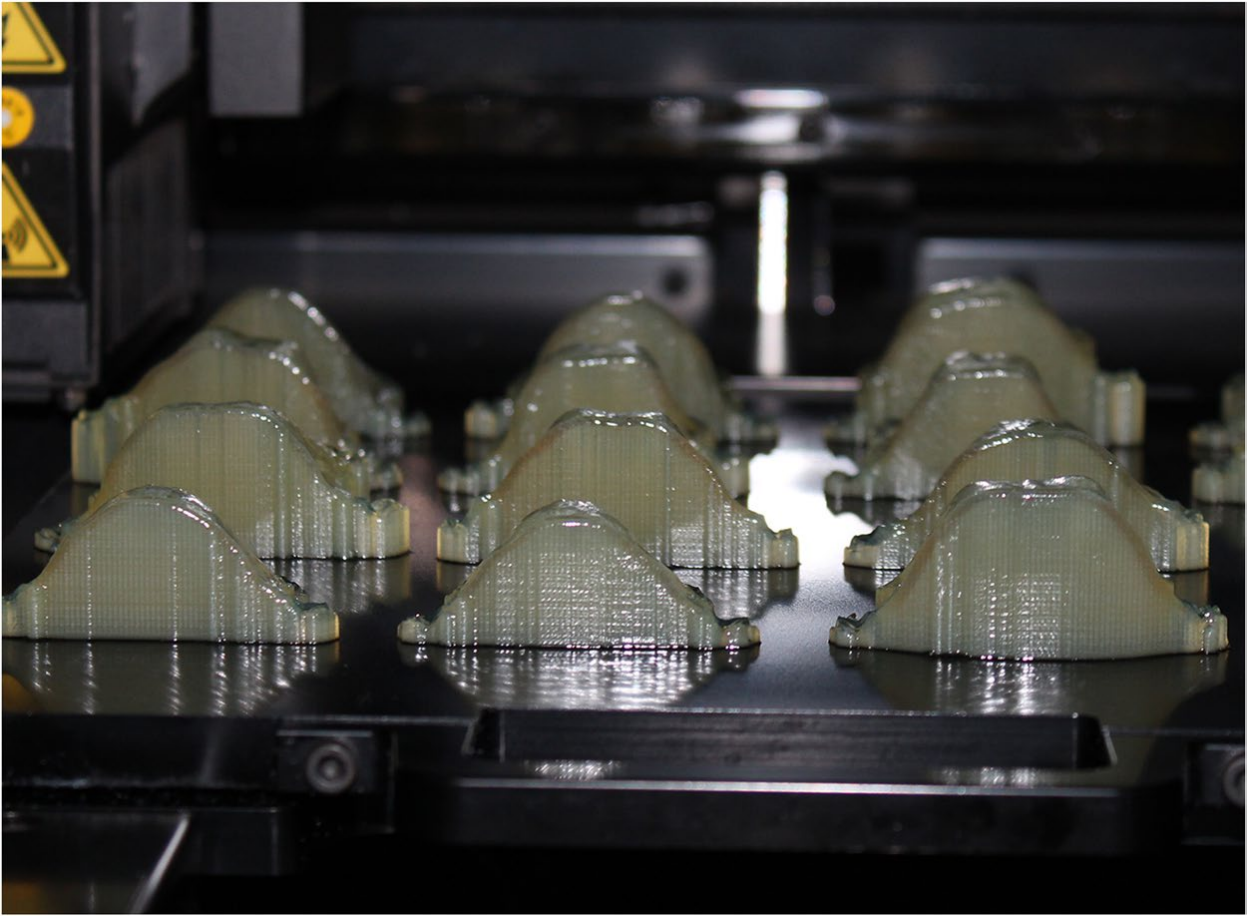
Major connectors manufactured by 3D printing machine with clear bio-compatible materials.

Once the design was finished, the virtual frameworks in STL file format were imported into 3D printing machine (Objet Eden 260VS Dental Advantage, Stratasys, U.S.A.). The layout of the virtual major connectors was implemented in the way that the occlusal rests were placed at the same level of the bottom and the major connectors were placed on top. So, the 3D printing procedure started from the occlusal rests and ended at the top of the palate plates. All the resin frameworks were created by Polyjet technology with a clear bio-compatible material (MED610, Stratasys, U.S.A.) (Fig. 2). MED610 is a rigid RP material with high dimensional stability and colorless transparency, which is medically approved for temporary in-mouth placement^25^. The major connectors overhung because of the shape of palatal vault, so the 3D printer jets a removable support material (Fullcure705, Stratasys, U.S.A.) under the smooth surfaces of major connectors for avoiding collapse during printing. After the resin frameworks were printed, the support materials were removed by flushing with high-pressure water gun easily.

### Adaptation Measurement

**Figure 3:**
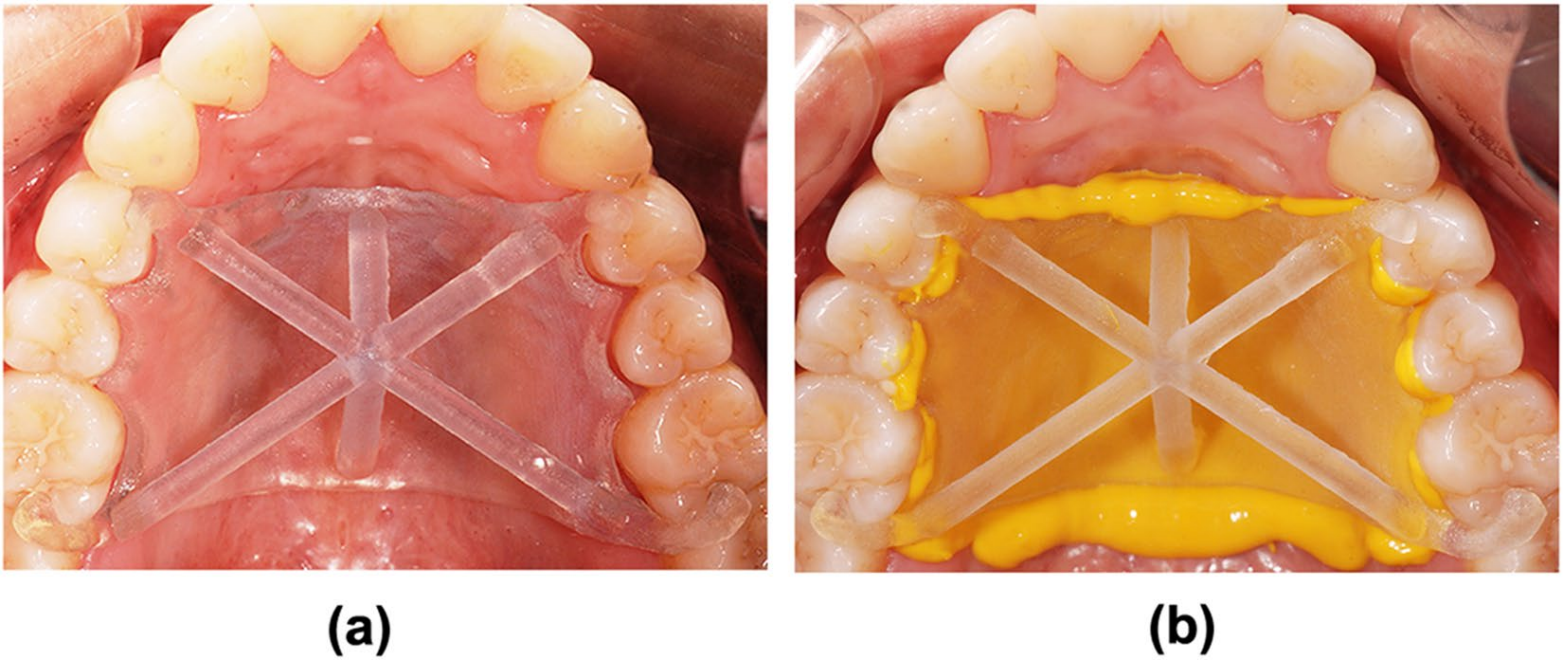
Place the major connectors in the mouth and measure the intraoral adaptation of the major connectors with light body silicone impression materials.

**Figure 4:**
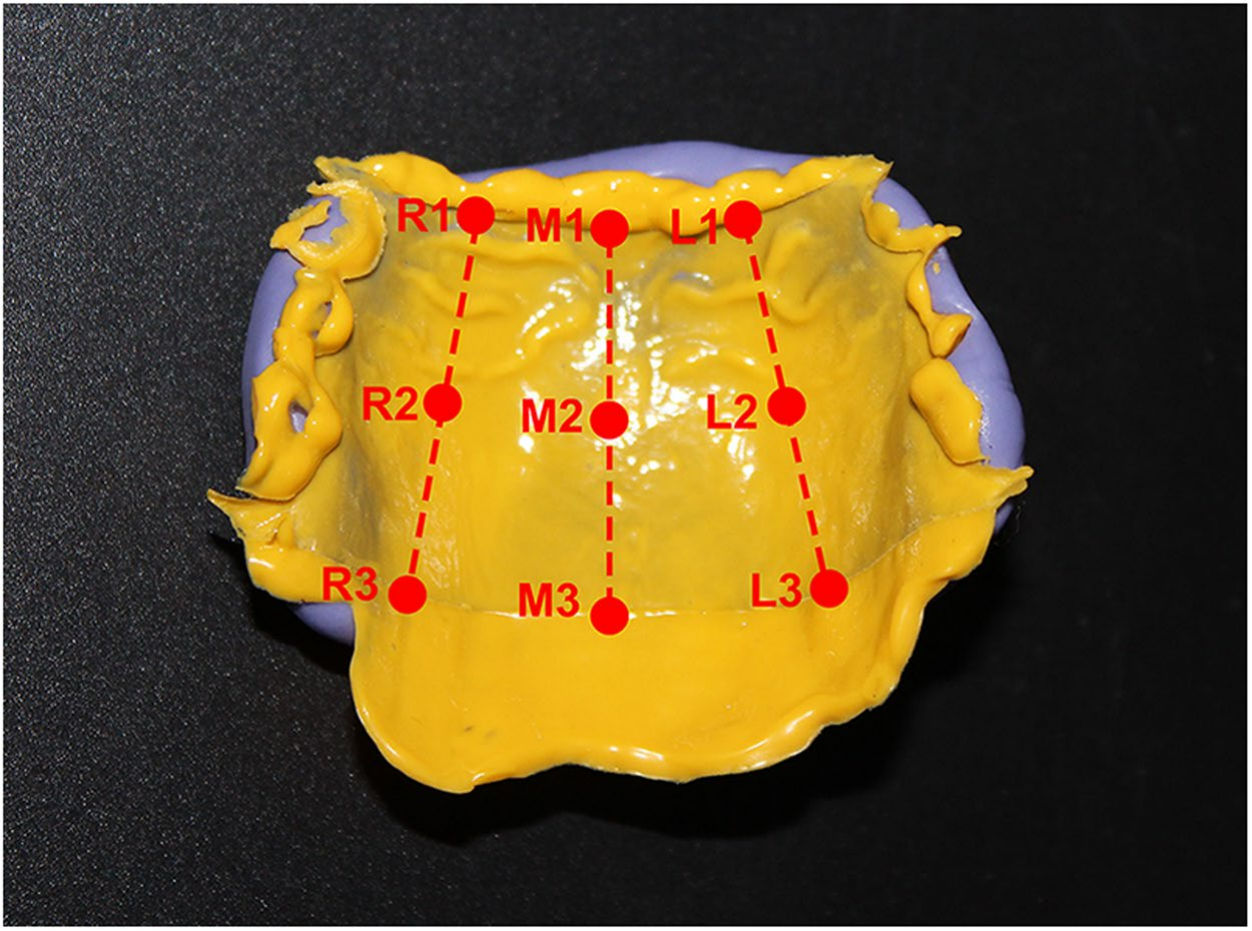
Landmark points on the layer of the polysiloxane impression material.

Before measuring the adaptation, the major connectors were firstly set in volunteers’ mouths to check seating. It was clinically accepted that proper adjustments could be carried out to achieve maximal stability and good seating of all the occlusal rests when needed^26,27^. The adaptation of the major connectors could be measured following that the framework was judged to exhibit good fit in the mouth. To measure the adaptation of the 3D printed major connector to the palatal soft tissues, light body silicone impression material (Honigum Light, DMG, Hamburg, Germany) was evenly syringed onto the tissue surface of the major connector, avoiding the flow of the material under the occlusal rests^26^. The major connector was set in mouth again and pressed onto volunteer’s upper jaw immediately (Fig. 3). The finger pressure was applied simultaneously and bilaterally on four occlusal rests during the entire setting procedure. After the light body silicone had set, the major connector and the light body silicone layer which remained on it were removed from the volunteer’s mouth. All above-mentioned operations were performed by a well-trained dentist and meanwhile assisted and supervised by the other experienced dentist. The silicone layer, representing the gap between the major connector and volunteer’s palatal soft tissues, were subsequently covered by application of a 5–10 mm thick heavy body silicone (Silagum Putty, DMG, Hamburg, Germany) for stabilization and contrast purpose. The silicone lays were segmented and measured at 9 landmark points:

Point M1, at the midpoint of the anterior border of the major connectors;

Point R1 (L1), at the midpoint between M1 and the rightmost (leftmost) of the anterior border;

Point M3, at the midpoint of the posterior border of the major connectors;

Point R3 (L3), at the midpoint between M3 and the rightmost (leftmost) of the posterior border;

Point M2, at the midpoint between M1 and M3;

Point R2 (L2), at the midpoint between R1 (L1) and R3 (L3) (Fig. 4).

The thickness of the silicone layers at each point was measured at ×30 magnification with a stereomicroscope (SteREO Discovery.V12 stereo microscope, Carl Zeiss, Germany). The recorded thickness for each point was an average of three readings.

### Data availability

The datasets generated during and/or analyzed during the current study are available in the Clinical Trial Management Public Platform, [http://www.medresman.org/pub/en/proj/search.aspx].

### Statistical analysis

All of the measurements and analyses were performed by one specialist at the clinic who was not involved in data collection and had no access to information that could identify individual volunteers. The statistical analysis was done with IBM SPSS Statistics Version 21 (IBM SPSS, Chicago, IL, USA). Statistical analysis was carried out using the Kolmogorov–Smirnov test for normal distribution and Levene’s test for homogeneity of variance. Paired two sample Student’s t-test was used to analyze the adaptation of the major connectors between Group I and Group E. The one-way Analysis of Variance (ANOVA) and LSD test were used to analyze the difference in adaptation at different parts of major connectors (among the midline palatine suture, the right and left sides of the palatal vault; or among the anterior margin, the middle section and the posterior margin) of the two groups. We set statistical significance at p < 0.05.

## Results

### Adaptation Measurement at Nine Landmarks

**Figure 5:**
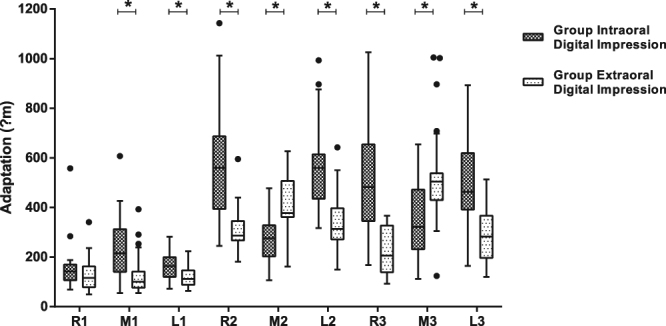
Boxplot of adaptation of the major connectors for Group I and Group E. The black dots represent outliers. Asterisk (*): p < 0.05.

The adaptation of major connectors ranged from 159.87 μm to 577.99 μm in Group I and 120.83 μm to 536.17 μm in Group E at nine landmarks (Table 1). The adaptation of major connectors in Group I was significantly different from that in Group E, except at Point R1 (p = 0.195) (Fig. 5). At Point M1, L1, R2, L2, R3 and L3, the silicon layers were thicker in Group I than Group E (p < 0.05), meaning the adaptation of major connectors derived from intraoral digital impressions was worse than that from extraoral digital impressions in these areas with adaptation difference of 44.11 μm to 286.85 μm. At Point M2 and M3, the silicon layers were thinner in Group I than Group E (p < 0.05), meaning the adaptation of major connectors derived from intraoral digital impressions was better than that from extraoral digital impressions in these areas with adaptation difference of 130.68 μm and 189.02 μm.

**Table 1:** The Adaptation of the Nine Landmark Points (Unit: μm).

		**Right(R)**	**Middle(M)**	**Left(L)**
**Anterior (1)**	Group I	159.87 ± 96.17	236.96 ± 126.23	164.94 ± 58.66
	Group E	129.50 ± 63.47	127.92 ± 84.03	120.83 ± 41.35
**Middle (2)**	Group I	577.99 ± 211.12	277.88 ± 87.25	563.53 ± 173.98
	Group E	309.56 ± 83.39	408.56 ± 115.86	340.88 ± 109.43
**Posterior (3)**	Group I	508.12 ± 220.16	347.15 ± 156.02	515.43 ± 179.27
	Group E	221.26 ± 94.74	536.17 ± 201.65	284.50 ± 106.30

### Adaptation Difference at Midline Palatine Suture and Two Sides of Palatal Vault

**Figure 6:**
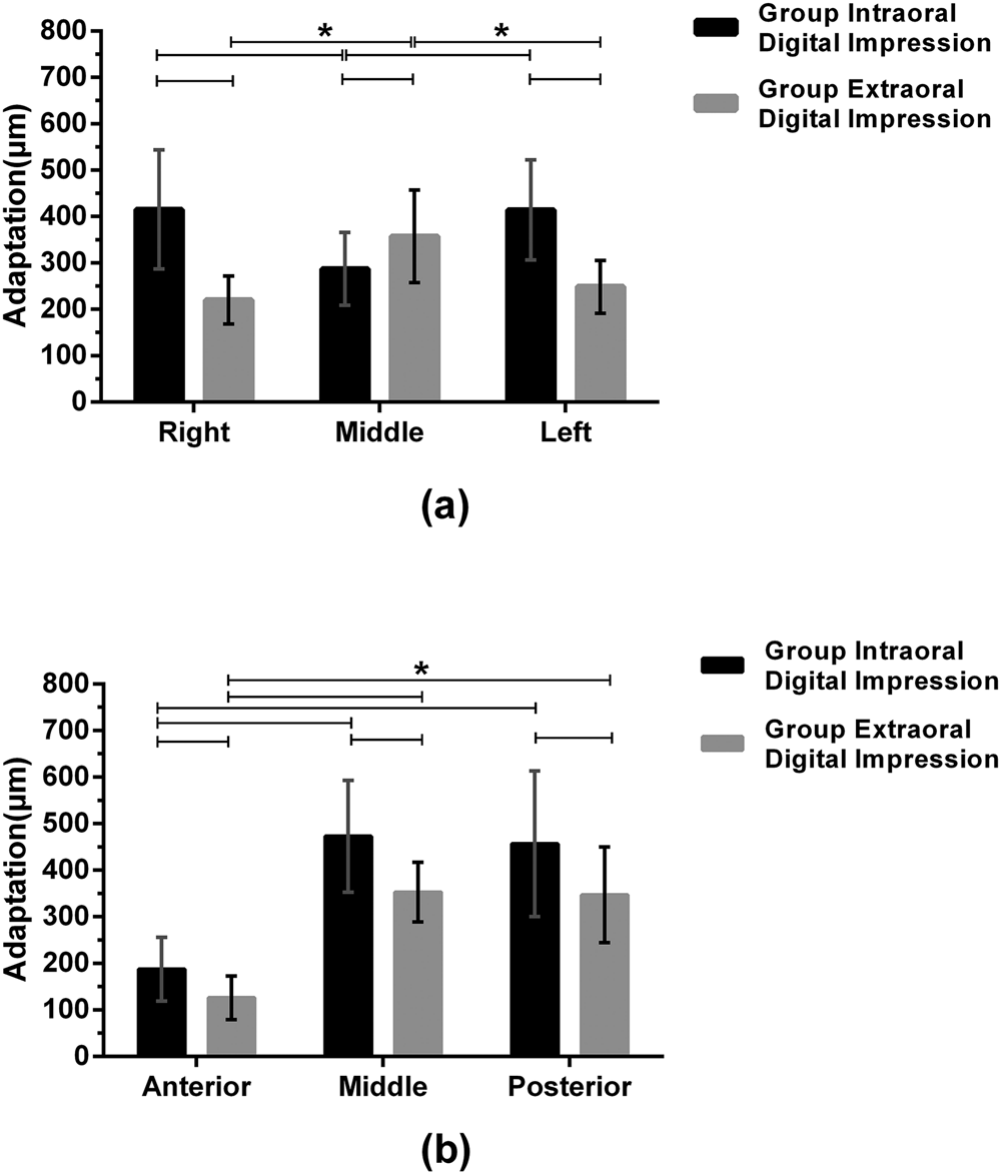
(**a**) Adaptation of the major connectors for Group I and Group E in the areas of midline palatine suture and the two sides of the palatal vault. (**b**) Adaptation of the major connectors for Group I and Group E at the anterior margin, the middle section and the posterior margin. Asterisk (*): p < 0.05.

The adaptation of major connectors at midline palatine suture, at left side and right side of palatal vault was (287.33 ± 78.04) μm, (414.63 ± 108.03) μm, (415.33 ± 128.07) μm in Group I and (357.55 ± 99.72) μm, (248.73 ± 56.92) μm, (220.11 ± 51.68) μm in Group E, respectively (Table 2). There was a significant difference in adaptation between Group I and Group E at midline palatine suture (p = 0.003), and the adaptation at this area was better in Group I than in Group E, (Fig. 6a). There was also a significant difference in adaptation between Group I and Group E at two sides of palatal vault (p < 0.001), but the adaptation at this area was worse in Group I than in Group E. In Group I, the adaptation of major connectors was better at midline palatine suture than at two sides of palatal vault (p < 0.001). No significant difference was found between the adaptation at left and right sides of palatal vault (p = 1). In Group E, the adaptation of major connectors was better at two sides of palatal vault than at midline palatine suture (p < 0.001). There was no significant difference between the adaptation at left and right sides of palatal vault (p = 0.208).

**Table 2:** Adaptation of the Major Connectors at the Midline Palatine Suture and the Two Sides of the Palatal Vault (Unit: μm).

	**Right**	**Middle**	**Left**
	**(R1 + R2 + R3)/3**	**(M1 + M2 + M3)/3**	**(L1 + L2 + L3)/3**
**Group I**	415.33 ± 128.07	287.33 ± 78.04	414.63 ± 108.03
**Group E**	220.11 ± 51.68	357.55 ± 99.72	248.73 ± 56.92
**(Group I) - (Group E)**	195.22 ± 123.61	−70.22 ± 104.32	165.90 ± 111.83
**p value**	<0.001	0.003	<0.001

### Adaptation Difference at Anterior Margin, Middle Section and Posterior Margin

The adaptation of major connectors at anterior margin, middle section and posterior margin was (187.63 ± 68.88) μm, (473.13 ± 119.90) μm, (456.90 ± 156.72) μm in Group I and (126.08 ± 47.20) μm, (353.00 ± 63.87) μm, (347.31 ± 103.13) μm in Group E (Table 3). There were significant differences in the adaptation at anterior margin (p = 0.002), middle section (p < 0.001) and posterior margin (p < 0.001) between Group I and Group E (Fig. 6b). The adaptation at anterior margin was better than that at middle section and posterior margin in both Group I and Group E (p < 0.001), and no significant difference in adaptation was found between middle section and posterior margin in both Group I and Group E (p = 0.970 and p = 0.994, respectively).

**Table 3:** Adaptation of the Major Connectors at the Anterior Margin, the Middle Section and the Posterior Margin (Unit: μm).

	**Anterior**	**Middle**	**Posterior**
	**(R1 + M1 + L1)/3**	**(R2 + M2 + L2)/3**	**(R3 + M3 + L3)/3**
**Group I**	187.63 ± 68.88	473.13 ± 119.90	456.90 ± 156.72
**Group E**	126.08 ± 47.20	353.00 ± 63.87	347.31 ± 103.13
**(Group I) - (Group E)**	61.55 ± 86.73	120.13 ± 107.10	109.59 ± 132.59
**p value**	0.002	<0.001	<0.001

## Discussions

The present study used a self-control method to compare the difference between adaptation of the major connectors fabricated from intraoral digital impressions and extraoral digital impressions respectively. The null hypothesis was partly rejected. It was feasible to manufacture the major connectors of RPDs by digital impressions and 3D printing techniques. But there was a significant difference in adaptation measurement between the major-connectors fabricated from the two different digital impressions.

It was found that there was space for a layer of light-body impression material under every major connector seated in mouth. The overall adaptation ranged from 159.87 μm to 577.99 μm of major connectors fabricated from intraoral digital impressions and 120.83 μm to 536.17 μm of major connectors fabricated from extraoral digital impressions. In one early study regard to conventional cast maxillary RPDs, the adaptation of the metal palatal plate ranged from 0.11 to 0.93 mm in mouth and from 0.09 mm to 0.68 mm for on the cast^26^. Diwan R. reported the mean adaptation was 0.64 ± 0.07 mm for the modified palatal plate and 0.56 ± 0.04 mm for the palatal strap on cast models, respectively^27^. There were inevitable gaps under major connectors for the reason of distortions of the wax patterns, dimensional changes in the refractory casts, contraction of the alloys and manmade errors of the technicians during the cast procedures of conventional RPD frameworks. The adaptation of major connectors found in the present study lay in the range of the results of the previous studies^26,27^, suggesting that the digital impressions of whole upper jaw could basically meet the clinical requirements for the adaptation of RPDs, and it was feasible to fabricate the major connectors of RPDs using intraoral or extraoral digital impression combined with 3D printing method. The results that the gaps under the major connectors fabricated from intraoral digital impressions were wider than those from extraoral digital impressions, which indicated that the conventional pressure impression was conducive to obtain better overall adaptation between major connectors and palatal soft tissues.

Obviously, there is a flexibility diversity of mucosae at different areas of palate. Thicker mucosae are at the palatal rugae and the two sides of the palatal vault with better flexibility, while thinner mucosae are at the center of palate, also namely hard areas, with worse flexibility. Vinyl polysiloxane materials are pressed onto jaws during the process of making conventional impressions, and oral mucosae at the two sides of the palatal vault will be oppressed more than the mucosa at hard areas. So, when further analyzing the difference in adaptation of CAD/CAM major connectors at different areas, it was found that the adaptation of major connectors fabricated from extraoral digital impressions were better at the two sides of the palatal vault than at the midline palatine suture. On the other hand, intraoral digital scan has no direct touch with the oral structure and it captures both the hard and soft tissues in a static state. In the present *in vivo* study, the adaptation of major connectors fabricated from intraoral digital impressions were found better at the midline palatine suture than at the two sides of the palatal vault. This phenomenon could be more likely attributed to the pressure of the impression materials between the major connectors and the resilient soft tissues^26,27^, so the gap might be widened at the two sides of the palatal vault.

The present study also showed a better adaptation at anterior margin than at middle section and posterior margin, and no significant difference in adaptation was found between middle section and posterior margin of the major connectors fabricated from both the intraoral and extraoral digital impressions. It indicated the results might be related with the technical characteristics of 3D printing. More errors could be found at those parts of the denture with steep slope and great curvature when manufactured by selective laser melting (SLM) technique^28^. The 3D printing technique used in this present study, PolyJet, could create models which was dimensionally similar to the virtual 3D STL image and showed adequate details with uniformly smooth surface^19,21,29^. But few researches have focused on its printing accuracy of objects with long span and large curvature. In preliminary experiments, the major connectors were printed from the top of the palatal plate to the occlusal rests, but there occurred to be deformations on resin frameworks so that a part of occlusal rests could not be fully seated on volunteers’ teeth. Thus, improvements were performed in the later experiments: adding supporting rods at the parts of large curvature on major connectors and applying a new policy that the major connectors were printed from the occlusal rests to the top of the palatal plate. The changes had solved the problem raised in preliminary experiments, and all the major connectors fabricated from digital impressions could achieve maximal stability and good seating on volunteers’ upper jaws.

Relatively slow development of CAD/CAM RPDs was also because of the higher demands with CAD/CAM equipments and techniques in order to meet the clinical requirements for large-sized oral prostheses fabrication. Dental System CAD software has specific modes for RPD designing which can simulate the conventional workflow in dental laboratory, that is: import of digital model data, surveying and determining the path of insertion, blockout of undercuts automatically, placing the retention grids, drawing the region for major connector, placing the minor connector and retainer (e.g., clasps and occlusal rests), sculpting (e.g., smoothing the surface of wax pattern and increasing or reducing the thickness of wax pattern), adding finishing line, placing the accessories (e.g., retentive posts, sprues and supporting rods), selecting the surface decorative figure of major connector, and export of designed frameworks data. The CAD software is able to provide much convenience for dentists and technicians to finish the designing of RPD frameworks as well as increase their work efficiency, however, it still exists some deficiencies at present such as lack of relief at specific areas and beading line at posterior margin of major connectors. During the conventional designing process of major connectors, relief should be placed at such areas as incisive papilla, maxillary hard areas and torus mandibularis to prevent pain of pressure then mucous injury under the function of occlusal force. Additionally, a 1mm-wide and 0.5 to 1mm-deep groove should be carved at the position corresponding to posterior margin of major connectors on plaster cast, forming a beading line to seal the boundary and reduce the foreign body sensation^14^. Thus, CAD software for RPDs needs to be improved and equipped with relevant function to adapt to the clinical demands better. Further development can also be based on the establishment of the 3D database for maxillary and mandibular soft tissues. When designing a RPD framework, the software can forecast the different flexibility extent of soft tissues at different regions and reduce the thickness of different soft tissues along with the normal direction in an automatic way, simulating the effect of pressure impressions.

The present study applied the biocompatible resin material to print major connectors for the research purpose. In clinic, there are two main methods to obtain CAM RPD frameworks: one is to print sacrificial patterns in wax or resin materials firstly and then cast to metal frameworks; the other is to directly manufacture metal frameworks by SLM technique. Direct manufacture is attempted with the aim of eliminating the time and material-consuming investment-casting process^16^, but is still of high production cost as well as propose demanding requirements for the property of printing machines and printing materials. All make the application of SLM technique in RPDs fabrication is not as wide as that in the fields of single crowns and FDPs.

## Conclusions

It is feasible to manufacture the major connectors of RPD by digital impression and 3D printing technique. Digital impressions of whole upper jaws can basically meet the requirements for RPDs fabrication in clinic, and both the adaptation of major connectors derived from the two kinds of digital impressions are clinically acceptable. Though the adaptation of major connectors derived from intraoral digital impressions is worse than that from extraoral digital impressions, intraoral digital impression has brought a new thought in the fabrication of RPDs. Further improvements in CAD softwares and CAM technique will lay the foundation of their application in the fields of RPDs fabrication. Feedbacks from patients about wearing comfort and usage satisfaction are also needed to instruct the clinical application in the future.
